# Genome-Wide Association Studies of Multiple Keratinocyte Cancers

**DOI:** 10.1371/journal.pone.0169873

**Published:** 2017-01-12

**Authors:** Luba M. Pardo, Wen-Qing Li, Shih-Jen Hwang, Joris A. C. Verkouteren, Albert Hofman, André G. Uitterlinden, Peter Kraft, Constance Turman, Jiali Han, Eunyoung Cho, Joanne M. Murabito, Daniel Levy, Abrar A. Qureshi, Tamar Nijsten

**Affiliations:** 1 Department of Dermatology, Erasmus MC Cancer Institute, Rotterdam, The Netherlands; 2 Department of Dermatology, Warren Alpert Medical School, Brown University, Providence Rhode Island, United State of America; 3 Department of Epidemiology, School of Public Health, Brown University, Rhode Island, United State of America; 4 National Heart, Lung, and Blood Institute's Framingham Heart Study, Framingham, Massachusetts, United State of America; 5 Population Sciences Branch, Division of Intramural Research, National Heart, Lung, and Blood Institute, National Institute of Health, Bethesda Maryland MD United State of America; 6 Department of Epidemiology, Erasmus MC University Medical Center, Rotterdam, The Netherlands; 7 Department of Epidemiology, Harvard T.H. Chan School of Public Health, Boston, MA, United State of America; 8 Department of Internal Medicine, Erasmus MC University Medical Center, Rotterdam, The Netherlands; 9 Program in Genetic Epidemiology and Statistical Genetics, Harvard School of Public Health, Boston, MA, United State of America; 10 Channing Division of Network Medicine, Department of Medicine, Brigham and Women's Hospital and Harvard Medical School, Boston, MA, United State of America; 11 Department of Epidemiology, Richard M. Fairbanks School of Public Health, Indiana University, Indianapolis, IN, United State of America; 12 Melvin and Bren Simon Cancer Center, Indiana University, Indianapolis, IN, United State of America; 13 Section of General Internal Medicine, Department of Medicine, Boston University School of Medicine, Boston, Massachusetts, United State of America; Harbin Medical University, CHINA

## Abstract

There is strong evidence for a role of environmental risk factors involved in susceptibility to develop multiple keratinocyte cancers (mKCs), but whether genes are also involved in mKCs susceptibility has not been thoroughly investigated. We investigated whether single nucleotide polymorphisms (SNPs) are associated with susceptibility for mKCs. A genome-wide association study (GWAS) of 1,666 cases with mKCs and 1,950 cases with single KC (sKCs; controls) from Harvard cohorts (the Nurses' Health Study [NHS], NHS II, and the Health Professionals Follow-Up Study) and the Framingham Heart Study was carried-out using over 8 million SNPs (stage-1). We sought to replicate the most significant statistical associations (p-value≤ 5.5x10^-6^) in an independent cohort of 574 mKCs and 872 sKCs from the Rotterdam Study. In the discovery stage, 40 SNPs with suggestive associations (p-value ≤5.5x10^-6^) were identified, with eight independent SNPs tagging all 40 SNPs. The most significant SNP was located at chromosome 9 (rs7468390; p-value = 3.92x10^-7^). In stage-2, none of these SNPs replicated and only two of them were associated with mKCs in the same direction in the combined meta-analysis. We tested the associations for 19 previously reported basal cell carcinoma-related SNPs (candidate gene association analysis), and found that rs1805007 (*MC1R* locus) was significantly associated with risk of mKCs (p-value = 2.80x10^-4^). Although the suggestive SNPs with susceptibility for mKCs were not replicated, we found that previously identified BCC variants may also be associated with mKC, which the most significant association (rs1805007) located at the *MC1R* gene.

## Introduction

Basal cell carcinoma (BCC) and squamous cell carcinoma (SCC) of the skin are known together as keratinocyte carcinomas (KC), since they both originate from keratinocytes of the epidermal layer of the skin, and share similar risk factors, treatments and prognosis [1]. KC is the most common cancer in adults of northern-European descent and is becoming a major health burden due to the high prevalence and increasing incidence in Western countries [2, 3]. A systematic review showed that patients with a primary BCC or SCC are likely to develop subsequent KCs with proportions as high as 44% in USA and 32% in The Netherlands [4]. However, it was recently shown that patients with only single KCs have a lower risk for subsequent KCs when compared with patients with a history of two or more KCs suggesting a differential risk profile of patients with single KCs than patients with a history of prior multiple KCs [3].

Environmental, tumour, and individual risk factors, including ultraviolet radiation (UVR), pale hair and skin, and male gender have been associated with an increased risk for multiple KCs (mKCs) [5–7]. There is also suggestive evidence for a genetic predisposition to mKCs, since genetic mutations in *PTCH1* [1, 8] and *PTCH2* [9] cause multiple BCCs [10] in individuals with nevoid BCC syndrome (NBCCS), a Mendelian disease. In addition, over 19 loci have been associated with sporadic BCC [11–13] and two with SCC. However, these previous studies included all prevalent non-melanoma skin cases and therefore it is not clear whether mKCs patients share the same genetic susceptibility variants as these with single KC.

In a recent study we found that common variants associated with BCC did not predict susceptibility for mBCC [14]. Other studies assessing genetic susceptibility in patients with mKCs are scarce. Here, we carried out a meta-analysis of GWAS on mKCs to investigate genetic susceptibility for mKCs comparing 1,241 mKCs to 2,822 single KCs (sKCs). We used patients with single KC as controls to increase the chance of identifying variants associated with susceptibility for having multiple KCs.

## Materials and Methods

### Study population

#### The nurses' health study (NHS), NHS II and the health professionals follow-up study (HPFS)–harvard cohorts

Study participants were included from three ongoing longitudinal cohorts: NHS, NHS II and HPFS. The NHS was established in 1976 when 121,701 married, female registered nurses aged 30–55 in the US were enrolled using a mailed questionnaire inquiring about their medical history and lifestyle practices. Between 1989 and 1990, blood samples were collected from 32,826 cohort members. NHS II began in 1989 when 116,430 female nurses aged 25–42 completed a mailed questionnaire. Between 1996 and 1998, blood samples were collected from 29,616 cohort members. The HPFS consisted of 51,529 male health professionals who completed their baseline questionnaire in 1986. Between 1993 and 1994, blood samples were collected from 18,159 cohort members. Information on lifestyle factors and medical history was collected biennially by mailed questionnaire. The follow-up rate exceeds 90% in each cohort. The study protocol was approved by the Institutional Review Board of Brigham and Women's Hospital and the Harvard School of Public Health.

We combined data from several case-control studies nested within the cohorts for type 2 diabetes (NHS and HPFS), coronary heart disease (NHS and HPFS), breast cancer (NHS and NHS II), colon cancer (NHS and HPFS), kidney stone (NHS, NHS II and HPFS), advanced prostate cancer (HPFS), endometrial cancer (NHS), gout (NHS and HPFS), glaucoma (NHS and HPFS), mammographic density (NHS), and pancreatic cancer (NHS and HPFS). The description of the studies is presented elsewhere [13].

**mKCs case ascertainment.** Participants reported diagnoses of cancers biennially. Medical records were reviewed to confirm the diagnoses. Medical records were not obtained for self-reported cases of BCC, but previous studies showed high validity of BCC self-reports [15, 16]. Information on the cumulative number of KCs was collected in 2004 (NHS), 2005 (NHS II) and 2008 (HPFS); details are presented elsewhere [5, 7]. A validation study among 200 cases who reported 5–10 and ≥11 KC showed a confirmation rate of 92% [7]. All the participants included in the analysis were Caucasians who reported at least one pathologically confirmed diagnosis of SCC or self-reported BCC in the cohort follow-up. For this study, cases were defined as individuals with more than one KC (mKCs) and controls were defined as those with single KC (sKCs).

#### The framingham heart study

The Framingham Heart Study (FHS) is a community-based prospective study that began in 1948 to characterize cardiovascular disease and its risk factors. The Original Cohort was composed of 5,209 Framingham residents primarily of white European-ancestry. In 1971, 5,124 offspring of the Original Cohort and their spouses were recruited into the Offspring Cohort. In 2002, 4095 children of the offspring cohort were invited to the Third Generation Cohort. The study design and participant descriptions of the three cohorts have been published elsewhere [17–19].

**mKCs case ascertainment.** Participants have undergone routine research examinations every two to six years. Cancer cases were identified at the research examinations or by medical history updates for participants who did not attend an examination. Two independent reviewers examined the medical records of all cancer cases and used the World Health Organization ICD-O coding and in 2010 ICD-10 coding to classify all primary tumours. All skin cancer cases were verified with pathology reports. FHS participants with GWAS genotype information and with pathologically confirmed skin cancer (until December 31 2013, melanoma excluded) were included in the current study. Participants with more than one KC were defined as cases and these with single KCs were defined as controls.

#### The rotterdam study (RS)

The RS is a prospective population-based follow-up study of the determinants and prognosis of chronic diseases, including skin cancer, in the elderly [20]. The RS consists of a major cohort (RS-I) and two extensions (RS-II and RS-III). RS-I started in 1990 and included 7,983 participants living in the Ommoord district (Rotterdam, the Netherlands). RS-II began in 2000 and now includes 3,011 participants. RS-III was started in 2006 and now includes 3,932 participants. By the end of 2008, the RS comprised 14,926 subjects aged 45 years or over. The RS consists predominantly (90%) of participants of North-European ancestry. A detailed description of the design of the RS is presented elsewhere [20].The Medical Ethics Committee of the Erasmus Medical Center and the review board of the Dutch Ministry of Health, Welfare and Sports have ratified the RS. Written informed consent was obtained from each participant.

**mKCs case ascertainment.** To identify histopathologically confirmed BCCs, SCCs and melanomas, RS participants were linked with the nationwide registry of histo- and cytopathology in the Netherlands (PALGA; up to 31st December 2013) [21]. The case definition for KC has been described previously [14]. In the majority of reports extracted from PALGA it was possible to distinguish between participants with single or subsequent tumours. If the diagnosis or the number of unique KC remained unclear, the medical files were searched by hand and a consensus decision was made. The number of KC was recorded separately for BCC and SCC. Individuals with either a single BCC or SCC were considered as controls while multiple BCC and/or SCC were taken as mKCs cases.

### Genotyping and imputation

Details of DNA collection, genotyping and quality control for the Harvard cohorts [13, 22], the FHS [23] and the RS [20] cohorts has been detailed elsewhere. A summary of the genotyping quality control for the all cohorts is presented in the supplementary file S1 Appendix.

#### Harvard GWAS

Genotyping was performed on three platforms: Affymetrix (n = 1230: 539 controls, 691 cases), Illumina HumanHap (n = 845: 363 controls, 482 cases), and Illumina Omni Express (n = 645: 287 controls, 358 cases). The genotypes per platform were merged from the different cohorts (NHS, NH II and HPFS) [13] and thus, had men and women. Based on combined GWAS genotypes on each genotyping platform and the 1000 Genomes Project ALL Phase I Integrated Release Version 3 Haplotypes (2010–11 data freeze, 2012-03-14 haplotypes) as reference panel, we imputed the genotypes of markers in the 1000 Genomes Project using MACHv.1.0.18.c [24]. Only SNPs with imputation Rsq > 0.95 and minor allele frequency (MAF) >1% were included in meta-analysis.

#### FHS GWAS

Genotyping was conducted using the Affymetrix 500K mapping array and the Affymetrix 50K gene-focused molecular imprinted polymer array. We imputed using 1000Genomes Phase I Version 3 as the reference panel using MACH-Minimac [24]. SNPs with MAF ≤1% and imputation quality value <0.3 were excluded.

#### RS GWAS

Details of genotyping approach is presented elsewhere [20]. Briefly, cohorts RS-I and RS-II were genotyped with the Infinium II HumanHap550K Genotyping BeadChip version 3 (Illumina, San Diego, California USA) and the cohort RS-II was genotyped using the Illumina Human 610 Quad Arrays. We imputed the RS-I, RS-II and RS-III cohorts separately, using 1000Genomes (GIANT Phase I version 3) as the reference panel and using MACH-Minimac with default parameters [24]. Next, markers with a MAF ≤1% and an imputation quality score (Rsq) < 0.3 were removed.

### Statistical analysis

#### Stage-1; discovery phase

The discovery samples (stage-1) consisted of the Harvard cohorts (NHS, NHS II, and HPFS) and the FHS cohort. The association analyses between the SNPs and mKCs were performed using an additive logistic regression model on subjects with more than one KC as cases and subjects with only one KC as controls. As the Harvard cohorts were genotyped on three different platforms [13], GWAS analyses were conducted for each platform, adjusting for age at first diagnosis of SCC/BCC, sex and four principal components of genetic variance (PCAs) using ProbABEL [25]. The association for each SNP from three platforms for the Harvard cohorts was combined in an inverse-variance-weighted meta-analysis using METAL [26].

The FHS GWAS was carried out using an additive generalized estimation equation (GEE) model [27] that takes into account the pedigree structure of the FHS study. The model was adjusted for age at first diagnosis, sex and four PCAs. These analysis were performed using the R package [27].

The quality control of the GWAS summary statistics from Harvard cohorts and the FHS GWAS summary statistics was performed using the EasyQC software [28]. After quality control there were 9,001,799 markers from Harvard cohorts and 8,246,930 markers from FHS. The cleaned files of both datasets (Harvard cohorts and FHS) were meta-analysed using the inverse variance approach implemented in METAL[26]. SNP heterogeneity was tested using I^2^ and Cochran’s Q, both of which are implemented in METAL. The inflation factor lambda (genomic control) was close to 1.0 (λ = 1.08) and therefore no further adjustments for genomic control were done The SNPs that showed significant associations with mKCs (p-value ≤ 5.5x10^-6^) were selected for stage-2 phase.

#### Stage-2 phase; replication and joint meta-analysis

The stage-2 analysis of the top SNPs identified in the discovery phase was carried out in the RS cohort. A logistic regression with an additive model to test for associations between SNPs and mKCs was implemented adjusting for age at diagnosis, sex and four PCs. The significance of the association was tested using the likelihood ratio test (LRT) with one degree of freedom. To correct for multiple testing, we calculated the pair-wise linkage disequilibrium (LD; r^2^) between the top SNPs using SNAP [29] and the p-values were adjusted by dividing the nominal p-value by the number of independent tests (SNPs were considered independent with r^2^≤0.6).

We also carried out a GWAS on the RS cohort as described previously [14] and used the p-values from the LRT [25] of the three cohorts for a meta-analysis. The quality control of the GWAS summary statistics per cohort was done with EasyQC [28]. After QC there were 7,898,815 markers. The cleaned files of the RS were then meta-analyzed with the FHS and Harvard cohorts using the weighted Z-score method, implemented in METAL [26]. SNP heterogeneity was tested using I^2^ and Cochran’s Q methods. The top SNPs were annotated to genes using Ensembl (http://browser.1000genomes.org/index.html).

## Results

The age and sex distribution of cases (mKCs) and controls (sKCs) for the stage-1 (discovery) and stage-2 cohorts are presented in Table 1. The discovery cohorts consisted of 1,666 subjects with mKCs and 1,950 subjects with sKCs. The ascertainment of KC was done primarily using self-reports in the Harvard cohorts. There were differences in the proportion of males in the cohorts, since the NHS and NHS II are women’s cohorts and HPFS is men’s cohort. We also observed that cases were older than controls.

**Table 1:** Demographic characteristics of the population-based cohort.

Cohorts	KC ascertainment		Cases (mKC)	Controls (sKC)	Sex(%male cases)	Sex(%male controls)	Median age^a^ cases (IQR)^b^	Median age^a^ controls (IQR)
**Stage 1**								
**Harvard**^c^*	Self-report		1,531	1,189	38.3	28.5	66 (59–73)	64 (58–71)
NHS	Self-report		920	817	0	0	64 (57–70)	66 (59–72)
NHS II	Self-report		23	33	0	0	45 (40–52)	50 (46–54)
HPFS	Self-report		588	339	100	100	67 (60–73)	69 (62–73)
**FHS**	Pathology records		135	761	60	50	66 (58–78)	66 (54–77)
**Stage 2**								
**RS combined**		Pathology records	575	872	40	50	73 (66–81)	69 (72–77)
RS1	Pathology records		345	542	43	52	78 (72–84)	74 (68–90)
RS2	Pathology records		142	178	53	52	68 (62–72)	70 (66–76)
RS3	Pathology records		88	152	39	36	57 (51–64)	60 (53–65)

FHS: Framingham Heart Study; mKC: multiple KCs; RS: Rotterdam Study; sKC: single KC.

^a^ Median age at first diagnosis

^b^ IQR: Inter-quantile range

^c^ Combined; dataset from the combined NHS, NHS II and HPFS cohorts.

*GWAS analysis for the Harvard cohorts were performed per GWAS platforms (see Materials and Methods) not per cohort.

**Fig 1:**
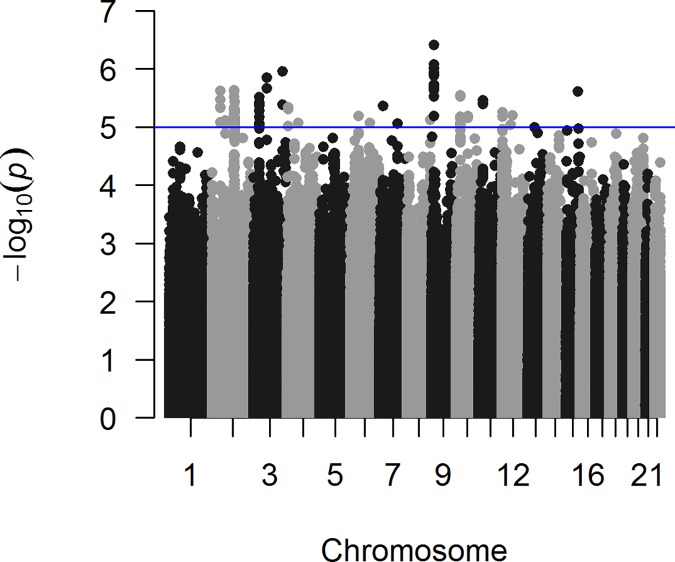
Manhattan plot of the GWAS associations for mKCs in the discovery sample (FHS and Harvard cohorts). The observed -log10 p-values (Y-axis) of the association between the SNPs and susceptibility for mKC are shown. All SNP are represented by dots and displayed per chromosome (X-axis).

In the discovery stage, suggestive genome-wide associations (p-value ≤5.5x10^-6^) were identified for 40 SNPs (Fig 1 and S1 Table). Due to the strong LD (r^2^>0.6) among these 40 SNPs (eight SNPs tagged 32 of the top SNPs), only eight of them were considered independent signals (S1 Table). The most significant hit was an intergenic SNP on the short arm of chromosome 9 (rs7468390, p-value = 3.92x10^-7^), with an OR (95% CI) of 0.73 (0.64–0.82) for the C allele (Table 2). This is a common SNP in strong LD with 13 other SNPs with suggestive associations (S1 Table and S1 Fig). The region of LD of rs7468390 spans approximately 13 kb. Of the 40 SNPs with suggestive associations, 29 were intergenic, three mapped to non-coding RNA, four within regulatory regions and four to the *NCKAP5* gene (S1 Table).

**Table 2:** Top SNPs identified in the discovery samples (Harvard cohorts and FHS) and p-values of the stage 2 in the RS and joint-meta-analysis stages (Harvard cohorts, FHS and RS- all combined).

					Stage1 (discovery phase)			Stage 2 (replication)		Combined analysis		
SNP id	A1^a^	A2^b^	Freq^c^	MAF^d^	OR^e^ e(95% CI)^d^	P-value	Direction^f^	Z-score^g^	P-value	Z-score ^h^	P-value	Direction^i^
**rs7468390**	**C**	**G**	**0.64**	**0.36**	0.73 (0.64–0.82)	3.92 x10^-7^	—	1.459	0.145	-3.300	9.69x10^-4^	—+
**3:171255288:ID**	**D**	**I**	**0.98**	**0.02**	3.10 (1.97–4.88)	1.11 x10^-6^	++	-0.898	0.369	3.551	3.84x10^-4^	++-
**rs58848026**	**T**	**C**	**0.85**	**0.15**	0.71 (0.62–0.82)	2.43x10^-6^	—	0.629	0.529	-3.664	2.48x10^-4^	—+
**rs4749296**	**T**	**G**	**0.36**	**0.64**	0.78 (0.70–0.87)	2.83 x10^-6^	—	-0.947	0.344	-4.358	1.31x10^-5^	—
**rs6803721**	**T**	**C**	**0.34**	**0.66**	0.77 (0.69–0.86)	3.03 x10^-6^	—	1.538	0.124	-2.964	3.04x10^-3^	—+
**rs4923076**	**A**	**T**	**0.52**	**0.48**	1.28 (1.15–1.41)	3.46 x10^-6^	++	-0.431	0.666	3.602	3.15x10^-4^	++-
**rs10167336**	**T**	**C**	**0.51**	**0.49**	1.27 (1.15–1.41)	3.70 x10^-6^	++	0.286	0.775	3.894	9.88x10^-5^	+++
**rs7799651**	**A**	**A**	**0.45**	**0.45**	0.73 (0.64–0.83)	4.37 x10^-6^	—	2.043	0.041	-2.553	0.011	—+

^a^A1: reference allele

^b^A2: other allele

^c^Frequency of A1

^d^Minor allele frequency of A2

^e^Odd ratios of A1. ORs and 95% confidence intervals (CI) were calculated from the weighted average of the effect size (regression coefficients and standard error) from the inverse-variance meta-analysis

^f^ Direction of the effect of A1 with +/- indicating a higher/lower disease risk for Harvard and FHS cohorts, respectively

^g^Z-scores from the replication

^h^Z-scores from the meta-analysis

^i^ Direction of the effect of the A1 with +/- indicating a higher/lower disease risk for Harvard cohorts, FHS and RS, respectively.

For the stage-2, we tested for associations between the 40 top SNPs from the stage-1 and mKC in an independent sample of 574 mKCs and 872 sKCs from the RS using an adjusted p-value of 0.006 (corresponding to a p-value of 0.05 divided by eight independent SNPs/tests. None of the SNPs replicated at this threshold (S2 Table). A combined analysis of both the stage-1 and stage-2 datasets showed suggestive associations for the 40 significant SNPs, but none reached genome-wide significance, and there was significant heterogeneity in the estimates, most likely due to the different direction of the effects in the RS [30] (Table 2 and S2 Table).

Other than the above-described 40 SNPs, we found suggestive statistical signals in the combined meta-analysis (p-values ≤5.5x10^-6^, S3 Table) for other SNPs. The most significant SNP was rs4761496 that mapped to an intergenic region of chromosome 12 (p-value = 4.5x10^-7^). Other SNPs with suggestive associations mapped to protein coding genes including *CSMD1* (rs11777268, rs116045237, rs17393453) and *PRF1* (rs35401316), both of which have an indirect involvement with SCC [31, 32].

Since up to 80% of KCs are BCCs, we also looked at whether SNPs previously associated with susceptibility for BCC conferred susceptibility to mKCs. Six of the 19 SNPs tested were significantly associated with susceptibility for mKCs (Table 3). However, only rs1805007, which that mapped to *MC1R* was significant (p-value = 2.8x10^-4^) after Bonferroni correction (adjusted p-value≤0.0026, 0.05/19). This gene is a well-known susceptibility locus for melanoma and KC. Other candidate loci identified in a recent GWAS for mBCC [14] were investigated but none were significantly associated with mKCs after Bonferroni correction (data not shown).

**Table 3:** Association analysis of BCC-loci and mKC susceptibility from the combined analyses (Harvard cohorts, FHS and RS).

SNP id	Gene	A1^a^	A2^b^	Freq^c^	MAF^d^	Z-score^e^	P-value	Direction^f^	I2^g^	ChiSq^h^
rs1126809	*TYR*	A	G	0.27	0.73	2.523	0.012	+++	0	1.97 (0.37)
rs4911414	20q11.22	T	G	0.35	0.65	0.892	0.373	+-+	0	1.65 (0.44)
rs1015362	20q11.22	T	C	0.28	0.72	-0.652	0.514	+—	52.9	4.25 (0.12)
rs7538876	*PADI6*	A	G	0.38	0.62	-0.049	0.961	-++	57.2	4.67 (0.10)
rs801114	1q42.13	T	G	0.7	0.3	0.109	0.913	++-	0	1.55 (0.46)
rs11170164	*KRT5*	T	C	0.08	0.92	0.639	0.523	-++	0	1.40 (0.50)
rs2151280	*CDKN2B-AS1*	A	G	0.47	0.53	-1.94	0.052	—	0	1.02 (0.60)
rs157935	*LINC-PINT*	T	G	0.70	0.3	0.991	0.322	+++	0	0.10 (0.95)
rs16891982	*SLC45A2*	C	G	0.05	0.95	-1.427	0.154	—	0	0.79 (0.67)
rs401681	*CLPTM1L*	T	C	0.43	0.57	-2.748	6.00x10^-3^	—	53.6	4.31 (0.12)
rs12210050	*EXOC2*	T	C	0.17	0.83	2.04	0.041	+++	0	1.20 (0.55)
rs7335046	*UBAC2*	C	G	0.87	0.13	1.569	0.117	+++	0	0.45 (0.80)
rs1805007	MC1R	T	C	0.08	0.92	3.633	**2.80x10**^**-4**^	**+++**	0	1.63 (0.44)
rs78378222	*TP53*	T	G	0.99	0.01	-1.689	0.091	-??	0	0.00 (1.00)
rs12203592	*IRF4*	T	C	0.17	0.83	2.37	0.018	+?+	0	0.19 (0.67)
rs12202284	*EXOC2*	A	C	0.21	0.79	2.224	0.026	+?+	0	0.00 (0.97)
rs8015138	*GNG2*	A	C	0.49	0.51	-1.3	0.194	-++	69.7	6.61 (0.04)
rs214782	*TGM3*	A	G	0.81	0.19	-1.953	0.051	—	0	0.81 (0.67)
rs7006527	*RGS22*	A	C	0.85	0.15	0.735	0.462	+++	0	0.08 (0.96)

^a^ A1:Reference allele

^b^A2:Other allele

^c^ Frequency of A1

^d^ Minor allele frequency of A2

^e^ Z-scores from the meta-analysis

^f^ Direction of the effect of A1 with +/- indicating a higher/lower disease risk for Harvard, FHS and RS cohorts, respectively

^g^ I^2^ statistic of the amount of heterogeneity

^h^ Cochran's Q-test statistics for heterogeneity with degrees of freedom equal to number of studies -1

Significant p-value after Bonferroni correction (adjusted p-value≤ 0.0026) is highlighted in bold.

## Discussion

In this two-stage GWAS of mKCs, we did not identify genome-wide significant associations between SNPs and mKCs. Several SNPs with suggestive associations mapped to genes involved in cancer pathology, but the findings need to be confirmed in larger samples. A candidate SNP-based analysis of previous BCC/SCC variants showed significant associations between mKCs and only one SNP (rs1805007) at *MC1R*, known to be associated with BCC, SCC and melanoma was significant. This suggests that genetic susceptibility for mKCs may partly overlap with that for BCC, which is expected given that up to 80% of KC are BCCs.

The lack of replication of the suggestive associations in might be due to several factors. First, phenotypic heterogeneity due to a differential ascertainment of KCs (pathology-confirmed versus self-reports) in the cohorts could have led to some phenotypic heterogeneity, although common variants for KCs have been replicated in the NHS, NHS II and HPFS cohorts [13] as well as in RS [14]. In addition, the ratio BCC/SCC may be different for the American and the European populations. In the RS, BCCs accounted for 82% of all mKCs. For the USA cohorts, BCC/SCC ratios were not available, but a higher proportion of SCCs after prior KCs in the USA were shown previously [4]. In addition, this study was underpowered to detect variants with small to moderate effects (S2 Fig). Indeed, in the joint meta-analysis it was shown that the eight variants with the most significant associations in the discovery samples had the opposite direction in the RS, which led to significant heterogeneity (Table 2). This may have caused a drop in the significance of the associations in the meta-analysis [30]. Interestingly, we found other SNPs hits in the joint analysis that had the same direction in the three cohorts, although the sample size was not large enough to reach genome-wide significance (lowest p-value was 4.5x10^-7^). Most likely, the lack of replication is a combination of both phenotype heterogeneity and low power to detect variants with moderate to low effects in the RS. Last but not least, one may argue that due to differences in the imputation quality thresholds between the Harvard cohorts and the RS and FHS, we may have missed GWAS hits. However, we did not expect a dramatic drop in power due to this reason because the Harvard cohorts, where a very stringent threshold was used to include SNPs for final meta-analysis (Rsq≥0.95) provided most of the markers (9,001,799 SNPS).

In the candidate SNP analysis nested within the GWAS, we found that *MC1R*, a gene previously associated with BCC was also associated with an increased risk for mKCs. This contrasts with a recent study from the RS where no association between known BCC-SNPs and susceptibility for mBCC was found [14]. Since the BCC cases were included in the replication dataset of this study, this shows that the previous findings were most likely due to a lack of power of the RS. Although only one of the 19 BCC-related SNPs was significant after Bonferoni correction, we found nominal associations for six of the previously identified BCC SNPs, suggesting that larger sample sizes will be necessary to validate these associations.

As shown previously [33], most variants identified through GWAS are expected to have low to moderate risks effects and therefore large consortia of participants with phenotype and GWA-SNP data are needed. While this is feasible for traits such weight or blood pressure for disease-related phenotypes this can be challenging. As mentioned above, all previous GWAS studies of BCC or non-melanoma skin cancer published so far did not separate cases with mKCs from those with sKC, and thus our series of mKCs cases could be considered as a rare phenotype. With our findings one may argue that there are no common variants with strong effects contributing to the genetic susceptibility for mKCs, although we only tested eight million common SNPs (frequencies higher than 2%). Whether the differential risk between patients with mKC and sKCs is due to genes or mostly due to environmental factors, or an interaction of genes and environmental factors remains to be elucidated. We did not test for SNP and environmental interactions that may be relevant in explaining susceptibility to mKCs, because we did not have all environmental risk factors assessed in all cohorts and the sample size was already small to detect SNP main effects. Heritability studies could help to determine to what extent genetic risk factors explain susceptibility for mKC. We found an heritability of 8% using GWAS data from the RS (data not shown), but the power was low to have a significant estimate. Determining the heritability for mKC as well as to identify individual susceptibility loci will require larger consortia of well characterized cases and controls. In addition, rare variants were not evaluated. Although such variants may not be clinically relevant to predict disease risk, they may reveal new pathways predisposing to mKCs and new targets for drug discovery, as in the example of vismodegib, a drug used to treat patients with NBCCS and sporadic, metastatic BCC [34].

## Conclusion

We found suggestive associations of common variants that were not replicated. To identify new loci and to confirm the suggestive associations found in this study, larger mKCs cohorts will be required.

## Supporting Information

S1 Appendix Quality control of the genotyping for the NHSI-II-HPFS, FHS and RS GWAS and supplementary references. (DOCX)

S1 Fig Regional plot of the most significant associations between SNPs in the short arm of chromosome 9 and mKCs. The plots represents the LD patterns of the most significant SNP in the study (rs468390) and nearby SNPs from this study (+/- 500 kb). Pairwise r2 is represented in colours. The log p-values of the associations of the rs468390 SNP and markers from the study is presented in the left Y-axis and the recombination rates is presented in the right Y-axis. The physical position of the markers is presented in Mb. The figure was generated using LocusZoom.
(TIF)

S2 Fig Power calculation of the study design. The power of the study was calculated using the program CaTS with sample size, p-value (1x10^-6^) and a disease prevalence of 10% as fixed parameters. An 80% power was expected for markers with MAF>25% and Odd ratios of >1.3
(TIF)

S1 Table Summary statistics of the most significant statistical associations between SNPs and mKCs in the discovery sample. The table presents the summary statistics of the eight regions with the most significant SNP associations and mKCs in the discovery sample.
(XLSX)

S2 Table Summary statistics of the most significant statistical associations from the discovery phase in the replication and joint meta-analysis phase. The table presents the summary statistics of the most significant associations that were followed in the replication sample and also in the combined analysis (meta-analysis).
(XLSX)

S3 Table Summary statistics of the most significant statistical associations for other SNPs and mKCs in the joint meta-analysis phase. The table presents the summary statistics of the most significant associations in the combined analysis (meta-analysis).
(XLSX)
