# Supplementary material for: Genome-Wide Association Studies of Multiple Keratinocyte Cancers
Source: PLoS One. 2017 Jan 12;12(1):e0169873. doi: 10.1371/journal.pone.0169873 (PMC5231365; doi:10.1371/journal.pone.0169873)
Supplement: S1 Appendix — (DOCX) [file pone.0169873.s001.docx]

**Supplementary Materials and Methods**

*Quality control of the NHSI-II-HPFS GWAS*

SNPs with deviations from Hardy-Weinberg equilibrium (HWE, p<10^-07^), call rates <95%, or MAF <1% were excluded. Samples with genotype call rates <= 95%, gender mismatch, non-European ancestry or outliers from the population sample were removed.

*Quality control of the FHS GWAS*

Quality control was performed by excluding samples with high heterozygosity (mean±3×s.d.), gender mismatch or sample call rates of <95%. SNPs with the following criteria were included for the GWAS: Hardy-Weinberg equilibrium test with a p-value >10^-06,^ MAF ≥1% and SNP call rate of ≥98%.

*Quality control of RS GWAS*

The quality control included the removal of SNPs with Hardy-Weinberg equilibrium deviations (p< 5x10^-06^), genotyping call rate <97%, gender mismatch and a high heterozygosity. We also Duplicates or first-degree relatives using identity-by-descent (IBD) estimates and outliers (three standard deviations away from the population mean) using multi-dimensional scaling (MDS) analysis with four principal components (PCs) were excluded.

**References**

1. Pruim RJ, Welch RP, Sanna S, Teslovich TM, Chines PS, Gliedt TP, et al. LocusZoom: regional visualization of genome-wide association scan results. Bioinformatics. 2010;26(18):2336-7. Epub 2010/07/17. doi: btq419 [pii]

10.1093/bioinformatics/btq419. PubMed PMID: 20634204; PubMed Central PMCID: PMC2935401.

2. Skol AD, Scott LJ, Abecasis GR, Boehnke M. Joint analysis is more efficient than replication-based analysis for two-stage genome-wide association studies. Nat Genet. 2006;38(2):209-13. Epub 2006/01/18. doi: ng1706 [pii]

10.1038/ng1706. PubMed PMID: 16415888.
