# Supplementary figures and images for: Genome-Wide Association Studies of Multiple Keratinocyte Cancers
Source: PLoS One. 2017 Jan 12;12(1):e0169873. doi: 10.1371/journal.pone.0169873 (PMC5231365; doi:10.1371/journal.pone.0169873)

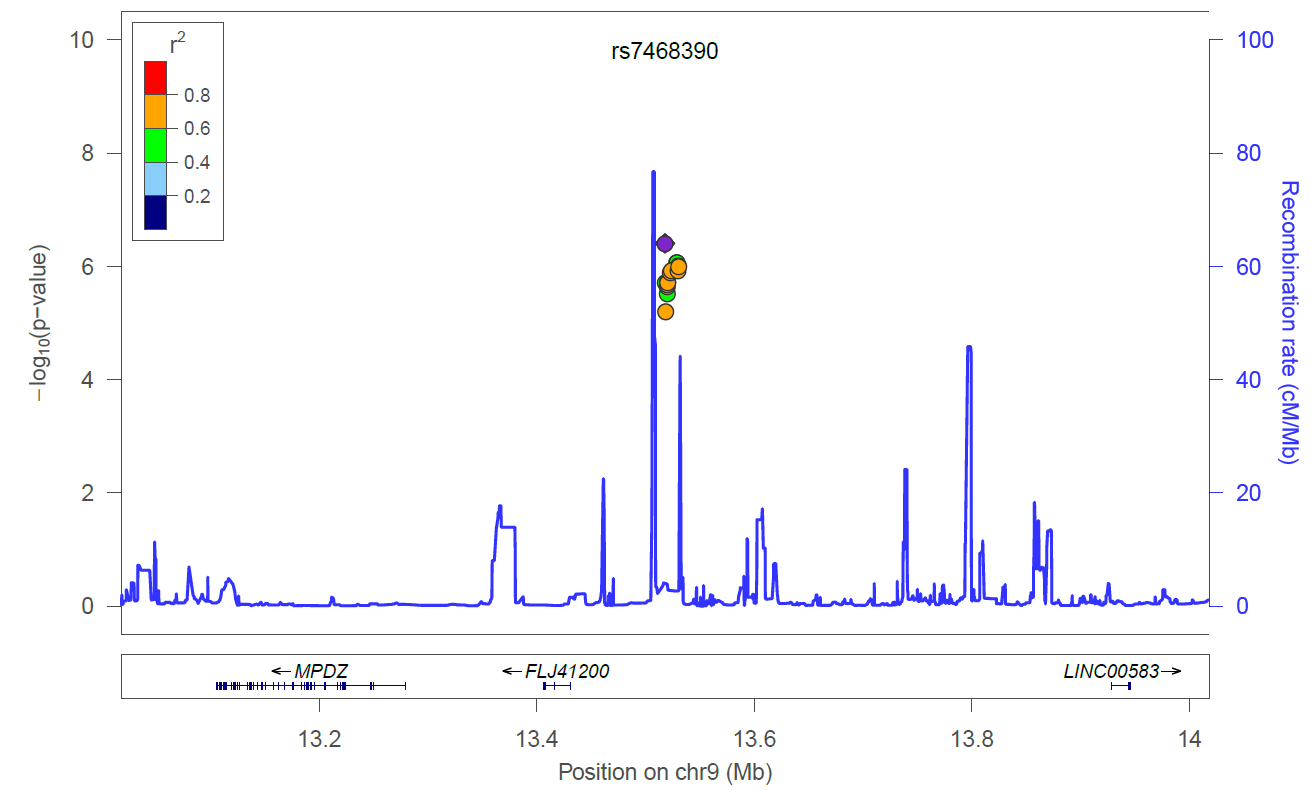

Supplement: S1 Fig — The plots represents the LD patterns of the most significant SNP in the study (rs468390) and nearby SNPs from this study (+/- 500 kb). Pairwise r2 is represented in colours. The log p-values of the associations of the rs468390 SNP and markers from the study is presented in the left Y-axis and the recombination rates is presented in the right Y-axis. The physical position of the markers is presented in Mb. The figure was generated using LocusZoom. (TIF) [file pone.0169873.s002.tif]

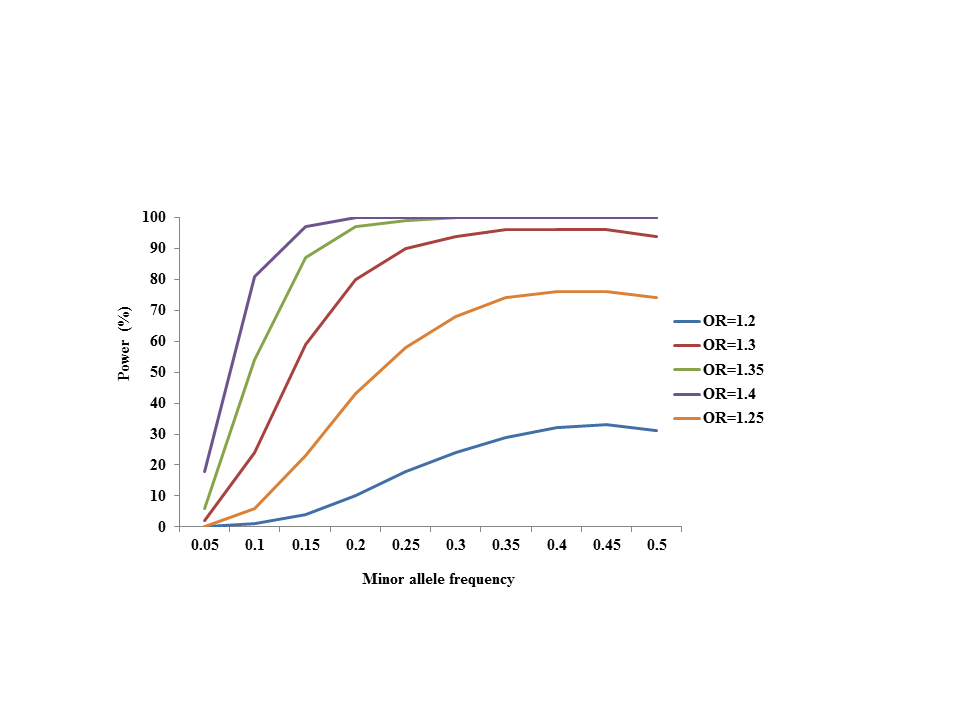

Supplement: S2 Fig — The power of the study was calculated using the program CaTS with sample size, p-value (1x10^-6^) and a disease prevalence of 10% as fixed parameters. An 80% power was expected for markers with MAF>25% and Odd ratios of >1.3 (TIF) [file pone.0169873.s003.tif]
